# The Molecular Signature More Than the Site of Localization Defines the Origin of the Malignancy

**DOI:** 10.3389/fonc.2019.01390

**Published:** 2019-12-17

**Authors:** Antonio Matrone, Liborio Torregrossa, Elisa Sensi, Daniele Cappellani, Walter Baronti, Raffaele Ciampi, Eleonora Molinaro, Clara Ugolini, Aleksandr Aghababyan, Luigi De Napoli, Francesco Latrofa, Gabriele Materazzi, Fulvio Basolo, Paolo Vitti, Rossella Elisei

**Affiliations:** ^1^Unit of Endocrinology, Department of Clinical and Experimental Medicine, Pisa University Hospital, Pisa, Italy; ^2^Anatomic Pathology Section, Department of Surgical, Medical, Molecular Pathology and Critical Area, Pisa University Hospital, Pisa, Italy; ^3^Unit of Endocrine Surgery, Department of Surgical, Medical, Molecular Pathology and Critical Area, Pisa University Hospital, Pisa, Italy

**Keywords:** cancer, thyroid cancer, lung cancer, mutation—genetics, molecular oncology

## Abstract

The diagnosis of the primary origin of metastases to the thyroid gland is not easy, in particular in case of concomitant lung adenocarcinoma which shares several immunophenotypical features. Although rare, these tumors should be completely characterized in order to set up specific therapies. This is the case of a 64-years-old woman referred to our institution for a very advanced neoplastic disease diagnosed both as poorly differentiated/anaplastic thyroid cancer (PDTC/ATC) for the huge involvement of the neck and concomitant lung adenocarcinoma (LA). Neither the clinical features and the imaging evaluation nor the tumor markers allowed a well-defined diagnosis. Moreover, the histologic features of the thyroid and lung biopsies confirmed the synchronous occurrence of two different tumors. The molecular analysis showed a c.34G>T (p.G12C) mutation in the codon 12 of K-RAS gene, in both tissues. Since, this mutation is highly prevalent in LA and virtually absent in PDTC/ATC the lung origin of the malignancy was assumed, and the patient was addressed to the correct therapeutic strategy.

## Background

Metastases to thyroid gland (TGM) from other primary tumors are rare entities and frequently reported in autoptic series (1, 2). The incidence of TGM widely varies in different series and has been related both to the site of the primary tumors and the ethnicity of the patients. TGM are more frequently caused by breast, lung, and kidney cancer (3–5). TGM has been reported also in case of gastrointestinal tract cancer, mainly in the Asian population (6, 7). The clinical presentation could be characterized by a rapid growth of a thyroid nodule with marked symptoms of dysphagia and/or dysphonia. In other cases, TGM show a clinical silent course without specific symptoms and were occasionally discovered after imaging procedures (neck US, CT scan, ^18fdg^PET-CT, etc.) during the assessment of the primary tumor (2, 4). Concomitant metastases in other organs are hardly ever present, and thyroid function is usually not compromised. Differential diagnosis with primary thyroid cancer may be done by fine needle aspiration cytology followed by immunocitochemistry evaluation. However, it is not easy to distinguish thyroid cancer from a thyroid metastasis particularly if derived from lung adenocarcinoma. Indeed, thyroid cancer and lung adenocarcinoma could have several common histopathologic features such as pale nuclei, finely granular chromatin, occasional nuclear grooves, and intra-cytoplasmic inclusions (2, 8, 9) that can make difficult a well-defined distinction between the two cancer entities even after the biopsy. Thus, in these cases, other techniques are required to perform a differential diagnosis that is crucial for choosing the best therapeutic strategy. We report a case of lung TGM for which we needed to turn to a next generation sequencing method to analyze specific molecular alterations to distinguish a primary thyroid cancer from a TGM.

## Case Presentation

A 64-years-old woman was referred to our institution for a second opinion about a very advanced neoplastic disease involving both the thyroid and the lung. The initial diagnosis was a poorly differentiated/anaplastic thyroid cancer (PDTC/ATC) with lung and bone metastases. The tumor mass was considered not surgically resectable because of the infiltration of the thyroid and cricoid cartilages, as well as of the carotid artery and the right jugular vein. An emergency tracheotomy with position of the 6 mm Shiley cannula because of the severe dyspnea, was performed. After the exclusion of contraindications and the placement of peripherally central catheter (PICC) in superior vena cava, a chemotherapy with Taxol (80 mg/m^2^) and Carboplatinum (AUC = 2) once weekly for 4 weeks, every 6 weeks, was performed and completed.

When the patient was referred to our Department, we discovered that she was a strong and old smoker (about 20 cigarettes daily for about 40 years). The physical examination showed hypotonic and hypotrophic muscles, and the presence of the enlargement of the neck with a large stiff mass; some palpable lymph nodes were evident in latero-cervical compartment, bilaterally. No other abnormalities were noted at physical examination except for the presence of tracheotomy. The patient was in medical therapy with atenolol for sporadic arrhythmia, steroids as anti-edema and omeprazole for gastric pain prevention.

**Figure 1:**
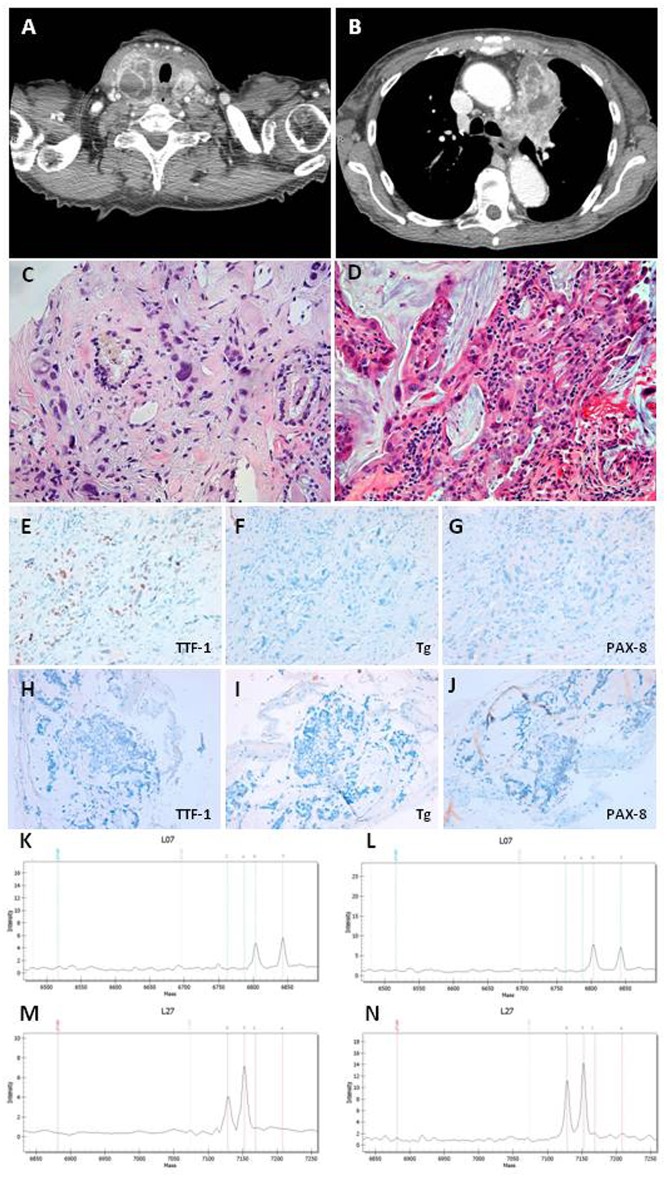
**(A)** CT scan section of thyroid neoplasia. **(B)** CT scan section of lung neoplasia. **(C)** Undifferentiated carcinoma with marked nuclear atypia intermixed to thyroid follicles in thyroid Tru-cut. Magnifications: x200. **(D)** Adenocarcinoma with mucinous differentiation in bronchial biopsy. Magnifications: x200. **(E–G)** Immunohistochemistry in thyroid Tru-cut showing focal immunoreactivity for TTF-1 and absence of immunoreactivity for Thyroglobulin and PAX-8 markers. Original magnifications: x200. **(H–J)** Immunohistochemistry in bronchial biopsy showing absence of immunoreactivity for TTF-1, Thyroglobulin, and PAX-8 markers. Original magnifications: x200. **(K,L)** Mass spectrometry of thyroid neoplasia with substitution c.34G>T (p.G12C) in the codon 12 of kRAS gene. **(M,N)** Mass spectrometry of lung neoplasia with substitution c.34G>T (p.G12C) in the codon 12 of kRAS gene.

To assess the neoplastic status of the patient, we performed a total body CT scan with i.v. contrast medium that showed the presence of multiple metastatic lymph nodes (max diam 17 mm in the right latero-cervical region) and a large thyroid gland with increased dimension in particular in the right lobe (Figure 1A). Moreover, the CT scan showed a large mass (9.0 × 4.5 cm) in the mediastinum, linked to the thyroid mass, involving the left upper lung lobe and occluding the left pulmonary artery branch (Figure 1B). Multiple metastatic lymph nodes were evident in paratracheal and paraesophageal regions. Two sclerotic lesions of L11 and D6 were also discovered.

Surgery was not indicated for the huge infiltration of the nearby vital structures. Radiotherapy counseling excluded the possibility to perform an external beam radiotherapy (EBRT) for the extension of the disease. Orthopedic counseling did not indicate any treatment for L11 and D6 lesions because there was no evidence of vertebral fractures. The only therapeutic option could be a systemic therapy, the most possible specific for the type of malignancy.

Serum tumor markers were all elevated and did not provide any specific information. In particular, Ca 125 was 35.9 U/ml (<35), Ca 15.3 was 169.3 U/ml (<25), Ca 19.9 was 59.3 U/ml (<39), CEA was 8.3 ng/ml (<5.2), CYFRA 21.1 was 8.8 ng/ml (<3.3).

Bronchoscopy showed the paralysis of left vocal cord with hypomobility of the right, arytenoids edema with reduced glottic space; the exploration of the bronchial tube showed the infiltration of the left upper lobe bronchus (LULB) with massive stenosis. Then, we performed a LULB brushing and a biopsy. To confirm the histological diagnosis, we decided to perform a Tru-Cut biopsy with 3 biopsies samples (2 left and 1 right) on the neck mass.

The thyroid Tru-Cut biopsy showed a poorly differentiated thyroid carcinoma (PDTC) with several anaplastic areas (Figure 1C). The immunohistochemistry for the thyroid specific proteins was TTF-1 focally positive, Tg, and PAX-8 negative (Figures 1E–G). The bronchus brushing showed the presence of an adenocarcinoma with widespread mucinous aspects, confirmed by biopsy (Figure 1D). The immunohistochemistry was CK7 positive, TTF-1, Napsin A, Tg, and PAX-8 negative (Figures 1H–J).

We than hypothesized that the patient could have two different types of cancer, PDTC/ATC of the thyroid and a mucinous adenocarcinoma of the lung. To solve this question, we decided to perform the molecular analysis of both the thyroid and bronchus biopsies.

The analysis was performed with a next generation sequencing method based on DNA extraction and MALDI-TOF mass spectrometry from paraffin-embedded tissues using CE-IVD validated kits “Myriapod Lung Status” on the “MassARRAY system” (Sequenom). Data were analyzed using the software “MassARRAY Analyzer 4” and “iGenetics Myriapod” (Diatech Pharmacogenetics). The analyzed genes were selected from a series of genes whose alterations are different in the two types of the tumors (Table 1) (10, 11).

**Table 1:** Genetic mutations of LA, PDTC, and ATC.

	**LA**	**PDTC**	**ATC**
EGFR^*^	14%	/	/
ALK^*^	/	4%	/
RET^*^	/	6%	/
MET	7%	/	/
nRAS^*^	/	21%	18%
hRAS	/	5%	6%
kRAS^*^	33%	2%	/
BRAF^*^	10%	33%	45%
PIK3CA^*^	7%	2%	18%
pTEN	/	4%	15%
EIF1AX	/	11%	9%
TERT^*^	/	40%	73%
NF1	11%	/	9%
TSH-R	/	2%	6%
STK11	17%	1%	6%
PAx8/PPAR gamma	/	4%	/
TP53	46%	8%	73%
ATM	/	7%	9%
RB1	4%	1%	9%
PI3K/AKT	/	11%	39%
SWI/SNF	/	6%	36%
HMTs	/	7%	24%
MMR	/	2%	12%
KEAP1	17%	/	/

LA, Lung Adenocarcinoma; PDTC, Poorly Differentiated Thyroid Carcinoma; ATC, Anaplastic Thyroid Carcinoma;

* *Genes analyzed in our samples after DNA extraction with MALDI-TOF mass spectrometry [EGFR (Exon 18, mutation and deletion of codon 709 and 719; Exon 19, mutation and deletion of codon 744–759; Exon 20, mutation and insertion of codon 767–775 and 790; Exon 21, mutation of codon 833, 835, 848, 854, 858, and 861); kRAS (Mutation codon 12, 13, 61); nRAS (Mutation in codon 12 and 61); BRAF (Mutation in codon 466, 469, 594, 597, 600); PIK3CA (Mutation in codon 542, 545, 1,043, 1,047); ALK (Mutation in codon 1,156, 1,196, 1,269); ERBB2 (Mutation in exon 20); DDR2 (Mutation in codon 239, 638, 768); MAP2K1 (Mutation in codon 56, 57, 67); RET (Mutation in codon 918); TERT (Mutation C228T)]*.

Both, thyroid (Figures 1K,L) and lung (Figures 1M,N) samples showed the substitution c.34G>T (p.G12C) in the codon 12 of K-RAS gene, while all the other mutations analyzed were negative. The patient was referred to the oncology unit who agreed with our hypothesis that the thyroid mass could be a metastasis of the lung adenocarcinoma. The patient was submitted to other 4 cycles of chemotherapy with the same scheme previously performed, but she died 3 months later.

## Discussion

There are some oncologic cases whose diagnoses are not easy because of the severe degree of de-differentiation of the tumor cells. This differential diagnosis is particularly difficult when a thyroid malignant lesion and a lung malignant lesion are simultaneously present in the same patient. Immunohistochemistry for some specific thyroid genes and corresponding proteins can be helpful, but we have to consider that thyroid transcription factor-1 (TTF-1) can be expressed by both thyroid cancer and lung adenocarcinoma (12) while thyroglobulin (Tg) is specific of thyroid cancer but, in PDTC, and even more in ATC cases, its expression is almost completely lost. Similarly, some serum tumor marker, such as CYFRA 21-1, could be elevated in the majority of lung carcinoma (13) but, although to a lesser extent, also in some cases of PDTC/ATC (14). Based on these considerations, neither the highly elevated values of serum tumor markers (e.g., CYFRA 21-1, CEA, Ca 125, Ca 15.3, or Ca 19.9) nor the positivity at immunohistochemistry of some thyroid specific genes (e.g., TTF-1 and PAX-8) were able to distinguish the two histologies.

Both thyroid cancer and lung adenocarcinoma can have mutations of genes involved in the MAP kinase pathways and some of them are almost exclusive of either lung adenocarcinoma or PDTC/ATC (Table 1) (10, 11, 15). It is indeed known that K-RAS is frequently mutated in lung adenocarcinoma and very rare or completely absent in PDTC and ATC, respectively (10, 11, 16). Moreover, the most common type of K-RAS mutations in thyroid cancer are Q61K, Q61R, and G12V (10, 17). It is worth noting that in several recent papers, which analyzed more than one thousand molecular profiles of PDTC/ATC by next generation sequencing, no K-RAS G12C mutation was found (10, 16, 18, 19). At variance, K-RAS p.G12C mutation is one of the most common type of K-RAS mutation in LA (11).

Currently, the presence of a K-RAS mutation identifies a subgroup of lung adenocarcinoma with a very poor prognosis (20), as confirmed by the very short survival of our patient.

Otherwise, in a prospective way, the importance of distinguishing the two tumors could be crucial to perform the right treatment. Indeed, the association of dabrafenib and trametinib demonstrated a robust clinical activity for the treatment of ATC with BRAF V600E mutation and was approved in USA from FDA (21). Also in case of LA, molecular targeted therapy are available and, the results from the phase III SELECT-1 trial including farnesyl transferase inhibition and synthetic lethality partners such as STK 33 showed promising biological activity against LA, in particular in p.G12C K-RAS mutated patients (22).

Moreover, in our case, not only the positivity of K-RAS mutation in both specimens, strongly supported the hypothesis that the thyroid mass could be a metastasis of the lung adenocarcinoma, but also its position in the lung lobe. The localization in the upper lobe is peculiar of the LA (23, 24), while PDTC/ATC metastases are usually multiple and located at the basis of the lung.

## Conclusions

This case represents a typical, although rare, case in which the diagnosis of the malignancy was based on the molecular signature more than the localization of the tumor mass and its immunohistochemical and biochemical features. The case is of interest since strongly demonstrates that nowadays the histology, especially in more complicated and not well-differentiated cases that can require specific therapies, needs to be associated with a molecular pathology analysis.

## Data Availability Statement

All datasets generated for this study are included in the article/supplementary material.

## Ethics Statement

The study was approved by Ethical Committee (CEAVNO—Comitato Etico Area Vasta Nord-Ovest). A written informed consent was obtained from the patient for the publication of this case report and any potentially-identifying information/images.

## Author Contributions

AM, PV, and RE contributed conception and design of the study. AM, DC, WB, FL, and EM collected clinical data and followed the patient during the clinical course. AA, LD, and GM performed the biopsies. LT, CU, and FB evaluated the histological specimens. ES performed the molecular analysis. RC contributed to the interpretation of the molecular data. AM and RE wrote the first draft of the manuscript. All authors contributed to manuscript revision, read, and approved the submitted version.

### Conflict of Interest

The authors declare that the research was conducted in the absence of any commercial or financial relationships that could be construed as a potential conflict of interest.
